# The quality of clinical practice guidelines in traditional medicine in Korea: appraisal using the AGREE II instrument

**DOI:** 10.1186/s13012-015-0294-1

**Published:** 2015-07-28

**Authors:** Tae-Young Choi, Jiae Choi, Ju Ah Lee, Ji Hee Jun, Bongki Park, Myeong Soo Lee

**Affiliations:** Medical Research Division, Korea Institute of Oriental Medicine, Daejeon, 305-811 South Korea; Liver and Immunology Research Center, Daejeon Oriental Hospital of Daejeon University, Daejeon, South Korea

**Keywords:** Clinical practice guideline (CPG), Korean traditional medicine, Appraisal of Guideline for Research and Evaluation (AGREE) II, Quality assessment, Evidence-based medicine (EBM)

## Abstract

**Background:**

This study aimed to evaluate the quality of the current clinical practice guidelines (CPGs) in traditional medicine (TM) in South Korea using the Appraisal of Guidelines for Research and Evaluation (AGREE) II instrument to further enhance the CPG development.

**Methods:**

A search was performed for guidelines in Korea from inception until March 2014 in the major Korean guideline websites [the Korean Medical Guideline Information Centre (KoMGI), the Korean Guideline Clearing House (KGC)], PubMed and seven Korean electronic databases; the Association of Korean Oriental Medicine (AKOM) was also consulted. Five independent assessors rated the quality of each CPG using the AGREE II instrument and calculated the mean score of each AGREE item. The overall agreement amongst reviewers was evaluated using the intra-class correlation coefficient (ICC).

**Results:**

Initially, 17 CPGs were examined for TM in Korea, and only 8 CPGs satisfied the inclusion criteria. The mean scores for each AGREE II domain were as follows: (1) scope and purpose, 60.0 % (CIs, 45.05-74.94 %); (2) stakeholder involvement, 56.11 % (41.28-70.94 %); (3) rigour of development, 42.7 % (23.48-61.92 %); (4) clarity and presentation, 62.50 % (50.89-74.10 %); (5) applicability, 20.31 % (13.96-26.66 %); and (6) editorial independence, 44.58 % (10.78-78.38 %). All of the CPGs were rated as “recommended with provisos or modifications”. The ICC values for CPG appraisal using the AGREE II ranged from 0.230 to 0.993.

**Conclusions:**

To improve clinical practice and health outcomes, well-developed CPGs are needed. The quality of CPGs for TM in Korea has remained suboptimal according to the AGREE II instrument evaluation. Therefore, guideline developers in Korea should make more of an effort to ensure high-quality CPGs.

**Electronic supplementary material:**

The online version of this article (doi:10.1186/s13012-015-0294-1) contains supplementary material, which is available to authorized users.

## Background

Clinical practice guidelines (CPGs) are *systematically developed statements to assist practitioner and patient decisions about appropriate health care for specific clinical circumstances* [1, 2]. CPGs have the potential to influence the care delivered by healthcare providers and the outcomes of patients [3]. Therefore, the quality of CPGs is critically important. Whereas guidelines were initially based on consensus amongst experts, guideline development has been gradually formalised and now consists of evidence-based guidelines [4]. Evidence-based CPGs ensure that the document or recommendation has been created using an unbiased and transparent process of systematically reviewing, appraising and using the best clinical research findings of the highest value to aid in the delivery of optimum clinical care to patients. Evidence-based CPGs can be used to develop quality measures and to support referrals when they are questioned by insurance companies; these CPGs also serve as education tools for patients.

The Appraisal of Guidelines for Research and Evaluation (AGREE) instrument was published by a group of international guideline developers and researchers [5]. The purpose of the AGREE is to provide a framework for assessing the quality of guidelines, provide a methodological strategy for the development of guidelines and inform what information and how information ought to be reported in guidelines [6]. Many countries have adopted the AGREE instrument to assess and validate the quality of CPGs, including CPGs for the management of particular diseases [7–10].

In Korea, developing clinical practice guidelines began approximately 10 years ago and resulted in increased interest in the development of CPGs in the healthcare community [11]. The Korean Medical Guideline Information Centre (KoMGi) [12], which opened in 2008, is a very useful nationwide dissemination tool of CPGs for health professionals. The Korean guideline clearinghouse (KGC) includes approximately 80 CPGs that have been developed by 40 Western Medical Associations in Korea. Although Western medicine has established quality evaluations of CPGs using the AGREE, the efforts to evaluate CPGs in Korea were not sufficient [13].

South Korea has maintained a dual healthcare delivery system that incorporates both traditional Korean and Western medicine. Traditional medicine (TM) in Korea remains in the beginning stage of development, and the use of CPGs is limited. Currently, only 17 CPGs are available regarding TM (Table 1). The CPGs are mixed with training manuals and textbook-like publications, and the aim of the CPGs is very unclear. The CPGs for TM have mostly been developed by the members of the Association of Korean Oriental Medicine (AKOM).

**Table 1:** Summary of developed guidelines in TM

No.	Condition	Intervention	Development group	Funding	Development method	Publishing year
1	Cervical vertebral portion	Acupuncture	AKOM	AKOM	Training manuals	2007
		Moxibustion				
		Chuna				
2	Lumbar vertebrae	Herbal medicine	AKOM	AKOM	Training manuals	2007
		Acupuncture				
		Moxibustion				
3	Obesity	Herbal medicine (*Ephedra sinica*)	The Society of Korean Medicine for Obesity Research	The Society of Korean Medicine for Obesity Research	Review	2007
4	Novel swine-origin influenza A (H1N1)	Herbal medicine	AKOM	AKOM	Training manuals	2009
5	Low fertility	Herbal medicine	AKOM	AKOM	Evidence-based CPG	2010
		Acupuncture				
		Moxibustion				
		Yoga…etc.				
6	Non-smoking	Acupuncture	AKOM	AKOM	Training manuals	2010
7	Whiplash injury-associated disorders (WAD)	Chuna	Korean Society of Chuna Manual Medicine for Spine and Nerves	Korean Society of Chuna Manual Medicine for Spine and Nerves	Training manuals	2010
		Exercise				
8	High blood hypertension	Herbal medicine	AKOM	AKOM	Text-like	2010
9	Diabetes	Herbal medicine	AKOM	AKOM	Text-like	2010
10	Cold	Herbal medicine	AKOM	AKOM	Text-like	2011
11	Hwa-byung (火病)	Herbal medicine	The Korean Society Of Oriental Neuropsychiatry, Hwa-byung Research C enter	Korean of Health and Welfare	Evidence-based CPG	2013
		Acupuncture				
		Moxibustion				
		Ect.				
12	Knee pain	Acupuncture	Korean Acupuncture and Moxibustion Medicine Society	Korean of Health and Welfare	Evidence-based CPG	2013
13	Low back pain	Acupuncture	Korean Acupuncture and Moxibustion Medicine Society	Korean of Health and Welfare	Evidence-based CPG	2013
14	Neck pain	Acupuncture	Korean Acupuncture and Moxibustion Medicine Society	Korean of Health and Welfare	Evidence-based CPG	2013
15	Atopic dermatitis	Herbal medicine	The Society of Korean Medical Ophthalmology, Otolaryngology and Dermatology	KIOM	Evidence-based CPG	2014
		Acupuncture				
		Pharmacopuncture				
		Moxibustion				
		Cupping				
		Etc				
16	Bell’s palsy	Herbal medicine	Korean Acupuncture and Moxibustion Medicine Society	KIOM	Evidence-based CPG	2014
		Acupuncture				
		Pharmacopuncture				
		Moxibustion				
		Cupping				
		Etc.				
17	Lumbar HIVD	Herbal medicine	The Korean Academy of Oriental Rehabilitation Medicine	KIOM	Evidence-based CPG	2014
		Acupuncture				
		Pharmacopuncture				
		Moxibustion				
		Chuna				
		Cupping				
		Etc.				

*AKOM* Association of Korean Oriental Medicine, *CPG* clinical practice guideline, *HIVD* herniated intervertebral disc, *KIOM* Korea Institute of Oriental Medicine

Compared to Western medicine, the clinical diagnosis and treatment in TM is less consistent, and the standards are poorer. In our previous study, which was a survey of Korean medical doctors concerning their perceptions of the development of CPGs for TM via e-mail, the results suggested the need to develop CPGs and to establish evidence in clinical practice and provide healthcare standards in TM [14]. It is necessary to evaluate the assessments of CPGs before developing these guidelines. In other words, a thorough understanding and investigation of the current status of CPGs is essential for quality management of CPGs in TM [15]. However, little is known about the quality of CPGs for TM. Therefore, the assessment of CPGs for TM is urgently required. To the best of our knowledge, no study has investigated the evaluation of CPGs of TM based on the AGREE instrument, although CPG development is regularly researched in Korea.

Considering these needs, this study aimed to investigate the current state of CPGs for TM through evaluating the quality of evidence-based guidelines in TM using the AGREE II instrument and to identify their quality to further enhance CPG development.

## Methods

### Study design

This study conducted a review of CPGs using the AGREE II instrument.

### Review protocol

This study was performed in accordance with the guidelines from the preferred reporting items for systematic reviews and meta-analyses (PRISMA) [16].

### Literature search

The CPG searches were conducted in March 2014 in major Korean guideline websites [the KoMGI and the KGC]. PubMed was searched as an international database, and KoreaMed, the Korea Institute of Science and Technology Information (KISTI), DBpia, the Korea National Assembly Library, the Korean Studies Information Service System (KISS) and the Oriental Medicine Advanced Searching Integrated System (OASIS) were searched as domestic databases.

Other sources such as contact with the AKOM and the Society of Korean Medicine were utilised to receive information on CPGs; additionally, the reference lists of all the obtained papers were searched. Hard copies of all the articles were obtained and read in full. The search terms were (clinical or practice or diagnosis or treatment or therapy or medication or drug or operation or prevention or management) and (guideline* or recommendation or consensus) and (traditional medicine or Korean medicine). The strategy of Korean search terms is shown in Additional file 1. Data regarding details such as development groups, financial source, development year and update status were collected for each document identified as a CPG (Table 1).

### Inclusion and exclusion criteria

The included CPGs in this study were original reports published in Korea that described TM interventions and provided sufficient methodological details based on evidence.

The inclusion criteria were as follows: (1) Korean language CPGs, which were produced by mainland Korea organisations, and (2) based on a systematic review of relevant research evidence. To determine whether the guidelines were evidence-based, we investigated whether they reported a search strategy, quality and data extraction that classified the evidence quality and graded the strength of the recommendation. If the CPGs had updates, only the most recent version was assessed.

CPGs were excluded if they met any of the following criteria: systematic reviews, narrative reviews, primary studies, critical pathways, training manuals for medical doctors, textbook-like publications, guidelines for patients, editorials, translations of foreign guidelines, secondary or multiple publications and short summaries.

### Appraisal of guidelines

The AGREE II instrument is a tool used to assess the methodological quality of evidence-based CPGs [5, 6]. It was translated into Korean to reduce inter-rater difference by Korean medical societies in 2011 [17]. The AGREE II consists of 23 items grouped into 6 domains and 1 overall assessment item: (1) scope and purpose (items 1–3)—the objective of the guideline, the target population and the health question; (2) stakeholder involvement (items 4–6)—involvement of stakeholders in the guideline development process and patients’ views and preferences; (3) rigour of development (items 7–14)—the process to collect and synthesise evidence and the recommendation development process; (4) clarity and presentation (items 15–18)—the language, structure and presentation of the guideline; (5) applicability (items 19–21)—evaluating the barriers and facilitators for the implementation and approach to improve uptake; and (6) editorial independence (items 22–23)—identifying biases resulting from competing interests. The overall assessment includes the rating of the overall quality of the guideline and whether the guideline would be recommended for use in practice. Each of the AGREE II items and the two global rating items were rated on a seven-point scale (1—strongly disagree to 7—strongly agree). A score was assigned depending on the completeness and quality of reporting. Domain scores were calculated by summing all the scores of the individual items in a domain and by scaling the total as a percentage of the maximum possible score for that domain. The scaled domain score was calculated as (obtained score − minimum possible score)/(maximum possible score − minimum possible score). The overall AGREE II evaluations (recommend, recommend with modifications or do not recommend each guideline) were independently determined by each assessor; then, consensus was achieved.

### Quality appraisal of guidelines

Each guideline should be assessed by at least two appraisers to increase the reliability of the assessment according to AGREE II. There were five appraisers with experience in the quality assessment of CPGs in this study who independently scored each guideline using the Korean translated AGREE II instrument. To improve the quality of the appraisal of guidelines, the CPG evaluation was pilot tested with the reviewers. All five appraisers, including a guideline methodologist, clinician and research assistant, received a training seminar and workshop that included education on the guideline development methods and the process and application of AGREE II. Additionally, we used the Korean-translated AGREE II. One study had examined the effect of the Korean AGREE II scoring guide and showed that the scoring guide reduced the inter-rater disagreement and improved the overall reliability [18].

### Investigation of heterogeneity

The scores of the five appraisers were used to calculate an average for each domain, and these scores were expressed as a percentage. Inter-rater reliability was calculated for each domain of the AGREE instrument using the intra-class correlation coefficient (ICC) with a 95 % CI. Statistical analysis was performed using SPSS for Windows 12.0 (SPSS Inc., Chicago, IL, USA).

## Results

### Guideline search and review process

**Fig. 1:**
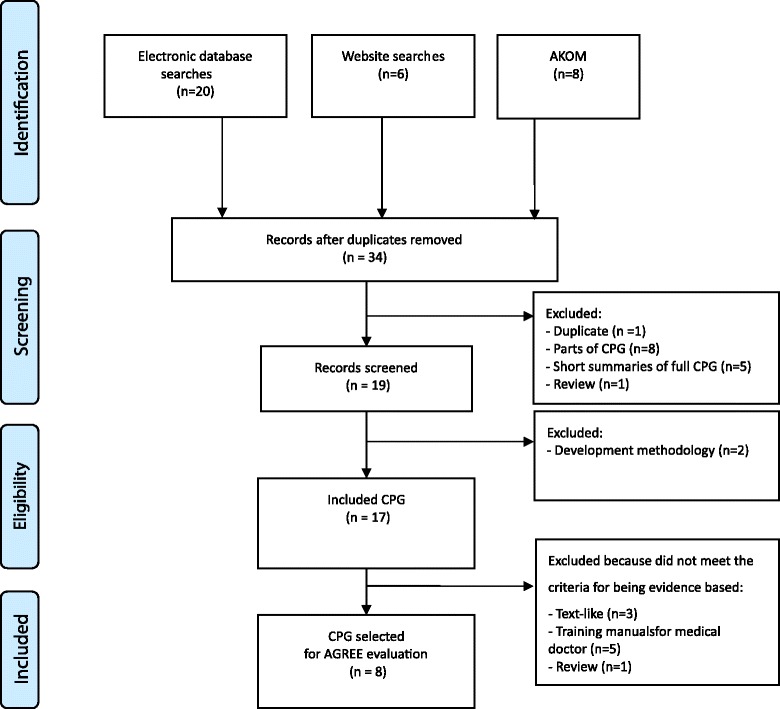
PRISMA diagram for the included studies. *AKOM* Association of Korean Medicine, *CPGs* clinical practice guidelines

A total of 34 articles were considered to be potentially relevant; after selection, a total of 17 guidelines were eligible (Table 1), and only 8 CPGs were selected based on a systematic review of the evidence. Nine guidelines were excluded because they were textbook-like (*n* = 4), training manuals for medical doctors (*n* = 4) or a review (*n* = 1) (Table 1). Finally, eight guidelines meeting our inclusion criteria were included, covering a period from 2010 to 2013 (Fig. 1 and Table 1).

### Guideline characteristics

A total of eight CPGs were eligible. The data from all of the included CPGs are listed. The eight CPGs focused on the following conditions: low fertility [19], Hwa-byung [20], knee pain [21], low back pain [22], neck pain [23], atopic dermatitis [24], Bell's palsy [25] and lumbar herniated intervertebral disc (HIVD) [26]. All eight CPGs were developed by academic societies. Specifically, the literal meaning of Hwa-byung is “anger disease” or “fire disease”, which is known as a culture-related syndrome related to anger in Korea [27]. In terms of funding, seven guidelines were supported by the Korean government, and another CPG reported receiving AKOM funding. Only one CPG was developed specifically for female adults, and the rest were for all adults. All CPGs stated that the recommendations were based on evidence. However, there was substantial variation in the grading systems of evidence quality and the recommendation strength (Table 2).

**Table 2:** Characteristics of eight evidence-based guidelines

Guidelines by medical condition [ref]	Target population	Method to formulate recommendations	Quality of evidence	Strength of recommendations
Low fertility [19]	Female adult	Based on SR of available evidence	Modified SIGN	Modified SIGN
		Consensus development based on evidence		
Hwa-byung (火病) [20]	Adult	Based on SR of available evidence	UMHS [43]	UMHS [43]
		Consensus development based on evidence		
		Based on available data		
Knee pain [21]	Adult	Based on SR of available evidence	NZGG grading system in accordance with the CAM	Clinical practice guidelines for acupuncture [44]
Low back pain [22]		Consensus development based on evidence	Clinical practice guidelines for acupuncture [44]	
Neck pain [23]				
Atopic dermatitis [24]	Adult	Based on SR of available evidence	Modified SIGN	Modified SING
Bell’s palsy [25]		Consensus development based on evidence	Modified Evidence-based guideline of clinical practice in Chinese medicine internal China	Modified Evidence-based guideline of clinical practice in Chinese medicine internal China
Lumbar HIVD [26]				

*CAM* complementary and alternative medicines, *NZGG* New Zealand Guidelines Group, *SIGN* Scottish Intercollegiate Guideline Network, *SR* systematic review, *HIVD* herniated intervertebral disc, *UMHS* University of Michigan Health System

### Appraisal of the AGREE II domains of the guidelines

The results of the assessments using the AGREE instrument are shown in Table 3 and Additional file 2.

**Table 3:** Scaled domain percentages for all appraisers for CPGs

Guidelines by medical condition [ref]	Scores, %						Overall assessment^a^
	Scope and purpose	Stakeholder involvement	Rigour of development	Clarity and presentation	Applicability	Editorial independence	
Low fertility [19]	45.56	36.67	22.92	53.33	12.50	1.67	R
Hwa-byung (火病) [20]	48.89	61.11	18.33	44.44	15.00	13.33	R
Knee pain [21]	45.56	41.11	31.25	55.56	16.67	21.67	R
Low back pain [22]	46.67	38.89	28.33	55.56	13.33	21.67	R
Neck pain [23]	48.89	44.44	30.83	55.56	17.5	20.00	R
Atopic dermatitis [24]	78.89	73.33	70.00	80.00	30.00	93.33	R
Bell’s palsy [25]	83.33	76.67	70.00	81.11	30.83	91.67	R
Lumbar HIVD [26]	82.22	76.67	70.00	74.44	26.67	93.33	R
Mean (95% CI)	60.00 (45.05-74.94)	56.11 (41.28-70.94)	42.70 (23.48-61.92)	62.50 (50.89-74.10)	20.31 (13.96-26.66)	44.58 (10.78-78.38)	–

*AGREE* Appraisal of Guidelines for Research and Evaluation, *CI* cofidence interval, *CPGs* clinical practice guidelines

^a^ *NR* not recommended, *R* recommended with provisos or modifications, *SR* strongly recommended, *U* unsure

#### Scope and purpose

This domain evaluates the overall objectives, expected benefits or outcomes and target population of the guidelines. The mean score for this domain was 60.0 % (CIs, 45.05-74.94). Three CPGs [24–26] scored >60 % (Table 3). In four CPGs [19, 21–23], the overall objectives were not specifically described.

#### Stakeholder involvement

This domain evaluates the degree of relevant professional group involvement, whether the views and preferences of the target population have been considered and whether the definition of target users has been clearly presented. The overall score in this domain was low with a mean of 56.11 % (CIs, 41.28-70.94). Three CPGs [24–26] scored >60 % (Table 3). Five CPGs [19–23] reported only the names and the institutional affiliation of the participants, and three CPGs [24–26] explicitly described the information about the area or discipline of the professionals. No CPG stated that the patients’ values or preferences were considered.

#### Rigour of development

This domain addresses the method of evidence search, grading, summary and the formulation of the recommendations. The mean score for this domain was 42.7 % (CIs, 23.48-61.92 %). Three CPGs [24–26] scored >60 % (Table 3). Most CPGs failed to demonstrate the association between evidence and the recommendations. By contrast, all the CPGs used systematic methods to search for evidence. Three CPGs [24–26] declared that they will be updated when new important evidence appears, whereas only four provided a timeline for updating.

#### Clarity and presentation

This domain generally evaluates the presentation and format of guidelines. The mean score was 62.50 % (CIs, 50.89-74.10 %), which was relatively higher than the other domains. Three CPGs [24–26] scored >60 % (Table 3). The key recommendations were easy to identify in most CPGs, but the clarity of the recommendations must be improved.

#### Applicability

This domain evaluates the consideration of facilitators or barriers to CPG implementation and monitoring criteria. The mean score of this domain was 20.31 % (CIs, 13.96-26.66 %), which was the lowest of all the domains. All the CPGs scored <60 %. All the CPGs failed to sufficiently consider applicability in guideline development.

#### Editorial independence

This domain addresses issues and competing interests of the guideline development members. The mean score was 44.58 % (CIs, 10.78-78.38 %). Three CPGs [24–26] scored >60 % (Table 3). Seven CPGs [20–26] reported receiving government funding, and only one CPG [19] reported receiving AKOM funding. Only three CPGs declared the potential conflicts of interest (COI) of the guideline developers.

#### Overall assessment

This assessment concerns “the rating of body quality of the guidelines and whether the guideline would be recommended for use in practice”. According to the appraisal of the individual domains and overall scores, all the CPGs were rated as “recommended with provisos or modifications”.

### Consistency

The ICC values for the AGREE II instrument appraisal are listed in Table 4. The ICC values, which indicate the overall agreement between reviewers, generally received higher reliability scores. The ICC values for CPG appraisal using the AGREE II ranged from 0.230 to 0.993. The ICCs for the AGREE appraisal conducted by the five raters were lowest in the “applicability” domain (0.230) but higher in the “scope and purpose”, “stakeholder involvement”, “rigour of development”, “clarity and presentation” and “editorial independence” domains (all >0.9).

**Table 4:** Inter-rater reliability for each quality domain

Domains	ICC (95 % CI)
Scope and purpose	0.956 (0.878–0.990)
Stakeholder involvement	0.940 (0.833–0.986)
Rigour of development	0.986 (0.962–0.997)
Clarity and presentation	0.904 (0.734–0.978)
Applicability	0.230 (−1.143–0.824)
Editorial independence	0.993 (0.981–0.998)

## Discussion

This study is the first to examine the quality of CPGs for TM in Korea using the AGREE II assessment tool. Our results showed that CPGs for TM are of moderate quality, which varies greatly between guidelines and across domains (Table 3). Currently, only eight evidence-based CPGs are available in TM in Korea. Thus, there have been few studies regarding CPGs, and little is known about their status. Particularly, the domains “rigour of development”, “applicability” and “editorial independence” were rated as low quality.

Three recently developed CPGs [24–26] received a strong score on the AGREE II. The development groups involved methodological experts who ensured that methodological checks were correctly applied and that the development process itself was fully documented. Therefore, methodological training should be established for guideline development groups to increase familiarity with guideline development standards such as the AGREE instrument and to incorporate these standards into CPGs.

Since 2006 in China, with the support of the World Health Organization/Western Pacific Regional Office (WHO/WPRO), multidiscipline panels were convened by the China Academy of Chinese Medical Sciences (CACMS) to develop the first collection of evidence-based CPGs in traditional Chinese medicine (TCM) [28–30]. These CPGs were also assessed using the AGREE instrument and showed a similar quality compared to the Korean CPGs for TM. In particular, evidence-based CPGs in TCM had the lowest score for applicability (27.09 %) compared to the other domains. Our AGREE II assessment also showed that the average score for applicability (20.31 %) was the lowest of the six domains. The applicability domain contains items about organisational barriers, resource implications for recommendations and key review criteria for monitoring and/or audit purposes. Most CPGs did not consider organisational barriers to CPG implementation and did not supply monitoring criteria to assess the CPGs’ effect.

Most domains showed a high reliability. Thus, inter-appraiser scores showed a strong correlation, and values were high for most domains except for the domain applicability. Because there were few or no implementation strategies or efforts to promote implementation in Korea, differences in awareness and the environment across appraisers are thought to affect inter-rater differences [13]. Future CPGs should suggest that special attention be paid to enhance the quality of applicability in developing evidence-based CPGs in TM.

In this study, all the CPGs used different grading systems to assess the quality of evidence and recommendations (Table 2). Specifically, there was a lack of strong relevance between the quality of evidence and the strength of recommendations, and many did not consider the consistency of results amongst the studies [31]. Consequently, many other organisations developed their own grading systems [28–30, 32]. Recently, the most widely used and known grading system is the Grading of Recommendations Assessment, Development and Evaluation (GRADE) [33]. The aim of this group is to develop a consolidated grading system for evidence quality and strength of recommendations to suggest that further CPGs use a comparable uniform grading system to evaluate the quality of evidence and strength of recommendations. However, there are obvious differences between the healthcare systems and clinical trial methods of medical systems [34, 35]. The methods for evaluating evidence and grading recommendations should be established according to the characteristics of TM medical literature [36, 37].

CPGs have been widely developed and support implementation with the aim of improving healthcare processes and patient outcomes, but the use of evidence-based practice remains haphazard. CPGs are both poorly developed and ineffectively implemented. To improve clinical practice and health outcomes, both well-developed CPGs and effective methods of CPG implementation are required [38]. Furthermore, there is a need to develop effective implementation methods to achieve large-scale adoption of proven innovations and recommended care [39].

High-quality systematic reviews (SRs) and randomised controlled trials (RCTs) are sources of the best evidence [40]. However, evidence-based CPG development is limited because of insufficient or conflicting evidence of high-quality SRs and RCTs in TM. Although CPG developers are frequently required to incorporate more than one form of evidence in their CPGs, information and guidance on how to achieve this goal are lacking. Formal consensus methods (i.e. the Delphi method, the nominal group technique, the RAND/UCLA Appropriateness Method (RAM) and the National Institutes of Health (NIH) consensus development conference) or guideline development are recommended [41]. CPGs that use informal consensus methods formulate recommendations without drawing on research evidence [42]. The consensus method is a basic method for developing CPGs in TM and requires further normalisation.

This review has a few limitations. Only a few CPGs regarding TM were found. It was assumed that CPGs that were not identified in this study have little potential use in practice. The CPGs evaluated in this report were primarily retrieved from the AKOM. The search results were examined in conjunction with a mail survey of seven clinical academic associations that are not member societies of the AKOM, which were added subsequently. Despite these limitations, this review is useful because it is the first to assess the current CPGs available in TM, and it evaluates the quality of their development process.

Several guidelines were produced in Korea within the last 5 years, but the overall quality was generally low. There are several recommendations for the future. First, an educational training programme should be developed for core confidence of guideline development groups to further familiarise them with guideline development standards such as the AGREE instrument and to incorporate these standards into CPGs. Second, budget preparation and the planning of financial support is required for developing and disseminating evidence-based CPGs, and a strategy for efficient partnership between the private sector and the government should be formulated and executed. Third, a methodology for the development of CPGs based on our TM environment, which lacks supporting evidence, is required, and technical support should be provided. Finally, future CPGs should use a consistent grading system to assess the quality of evidence and strength of recommendations.

In conclusion, the quality of CPGs for TM in Korea has remained suboptimal according to the AGREE II instrument evaluation. Specially, the use of AGREE II in the development process ensures that these considerations are incorporated and must make an increased effort to ensure the high quality of CPGs. Therefore, guideline developers should consider adopting evidence-based CPGs for developing trustworthy guidelines to ensure the translation of evidence into practice.

## Additional files

Additional file 1:
**Search terms used in titles and abstracts.**
Additional file 2:
**The individual scores of the appraisers.**

## Abbreviations

AGREE: Appraisal of Guidelines for Research and Evaluation
AKOM: Association of Korean Oriental Medicine
CACMS: China Academy of Chinese Medical Sciences
COI: conflicts of interest
CPG: clinical practice guideline
EBM: evidence-based medicine
GRADE: Grading of Recommendations Assessment, Development and Evaluation
HIVD: herniated intervertebral disc
ICC: intra-class correlation coefficient
KGC: Korean Guideline Clearing House
KISS: Korean National Assembly Library, Korean Studies Information Service System
KISTI: Korea Institute of Science and Technology Information
KoMGI: Korean Medical Guideline Information Center
NIH: National Institutes of Health
OASIS: Oriental Medicine Advanced Searching Integrated System
PRISMA: preferred reporting items for systematic reviews and meta-analyses
RCT: randomised controlled trial
SR: systematic review
TM: traditional medicine
WHO/WPRO: World Health Organization/Western Pacific Regional Office
