# Supplementary material for: The quality of clinical practice guidelines in traditional medicine in Korea: appraisal using the AGREE II instrument
Source: Implement Sci. 2015 Jul 28;10:104. doi: 10.1186/s13012-015-0294-1 (PMC4515911; doi:10.1186/s13012-015-0294-1)
Supplement: Additional file 1: — Search terms used in titles and abstracts. [file 13012_2015_294_MOESM1_ESM.pdf]

Additional file 1. Search terms used in titles and abstracts

	Korean search term
#1	임상 or 진료 or 진단 or 치료 or 약물 or 예방 or 관리
#2	지침 or 권고 or 합의
#3	한의학 or 전통의학
#4	#1 and #2 and #3
