# Supplementary material for: The quality of clinical practice guidelines in traditional medicine in Korea: appraisal using the AGREE II instrument
Source: Implement Sci. 2015 Jul 28;10:104. doi: 10.1186/s13012-015-0294-1 (PMC4515911; doi:10.1186/s13012-015-0294-1)
Supplement: Additional file 2: — The individual scores of the appraisers. [file 13012_2015_294_MOESM2_ESM.pdf]

Additional file 2. The individual scores of the appraisers

Domain	Guideline *	CPG 1					CPG 2					CPG 3					CPG 4					CPG 5					CPG 6					CPG 7					CPG 8				
	**Appraiser**	1	2	3	4	5	1	2	3	4	5	1	2	3	4	5	1	2	3	4	5	1	2	3	4	5	1	2	3	4	5	1	2	3	4	5	1	2	3	4	5
**Scope and purpose**	Item 1	4	4	5	5	6	4	5	5	5	6	4	5	5	5	6	6	6	7	6	4	4	3	3	4	5	6	6	7	7	5	6	6	7	7	5	6	7	6	7	5
	Item 2	3	3	3	2	3	3	3	3	2	3	3	3	3	2	3	3	2	3	2	3	3	2	3	4	2	6	6	7	4	5	6	5	7	5	6	7	6	7	5	6
	Item 3	4	4	4	2	4	4	4	4	4	4	4	4	4	2	4	4	4	4	2	3	5	4	5	5	4	6	6	7	6	5	6	5	7	5	4	6	5	7	5	5
**Stakeholder involvement**	Item 4	4	4	3	5	4	4	4	3	5	4	4	4	3	4	4	5	5	4	6	4	4	4	2	2	4	6	7	7	7	7	6	7	7	7	7	6	7	7	7	7
	Item 5	3	4	1	3	2	3	4	1	5	2	3	4	1	5	2	5	5	3	6	5	1	2	1	1	1	4	4	3	3	4	3	4	3	3	2	4	5	3	4	3
	Item 6	4	5	3	4	3	4	6	3	4	3	3	4	3	3	3	6	5	6	4	3	6	7	3	3	7	6	6	7	7	6	6	5	7	7	6	6	6	6	7	6
**Rigor of development**	Item 7	5	5	5	5	4	5	5	5	4	4	5	5	5	5	3	2	2	2	1	1	5	5	4	4	4	6	7	6	5	6	6	5	5	7	6	6	7	6	4	6
	Item 8	4	4	1	4	2	3	4	1	4	2	3	4	1	4	2	1	2	2	1	3	1	2	1	1	2	5	6	7	5	5	5	6	7	7	6	5	6	7	4	5
	Item 9	4	6	5	1	6	3	3	5	1	5	3	3	5	1	5	1	1	3	1	5	2	2	5	5	5	6	5	7	6	6	6	5	7	7	6	6	6	7	6	6
	Item 10	3	3	1	2	3	3	2	1	2	3	3	1	1	2	3	1	1	1	1	3	1	1	1	1	3	5	5	4	3	5	5	5	4	3	5	5	5	5	6	5
	Item 11	3	3	3	4	3	4	5	3	4	3	3	5	3	4	3	4	5	4	1	1	3	2	3	4	2	4	4	6	5	4	3	4	6	4	4	4	4	7	4	4
	Item 12	1	1	1	3	1	1	1	4	4	1	1	1	1	3	1	2	2	4	2	4	1	1	3	4	4	3	6	7	5	6	3	5	7	5	6	5	6	7	5	5
	Item 13	3	2	3	3	2	3	2	3	3	2	3	2	3	3	2	3	3	2	4	3	2	2	1	1	2	5	7	4	3	2	5	7	4	3	2	5	7	4	3	2
	Item 14	1	1	1	1	2	1	1	1	1	2	1	1	1	1	2	1	1	1	1	1	1	1	1	1	1	6	6	5	5	5	6	6	5	5	5	6	6	5	5	5
**Clarity and presentation**	Item 15	4	3	3	2	5	3	4	3	3	5	3	4	3	2	5	3	2	3	3	3	3	2	3	3	5	6	6	7	4	6	5	6	7	5	5	6	6	7	5	6
	Item 16	4	4	4	4	3	4	3	4	4	3	5	3	4	4	3	5	6	1	3	4	5	3	5	5	4	5	5	3	3	3	4	5	5	6	4	5	5	4	5	4
	Item 17	6	6	7	4	6	6	6	7	4	6	6	6	7	4	6	5	5	6	3	3	5	2	6	6	6	7	7	7	7	6	7	7	7	7	7	7	7	7	7	7
**Applicability**	Item 18	3	3	3	1	4	1	1	3	5	4	1	1	3	1	4	1	1	3	6	1	1	1	3	4	6	2	5	4	3	3	3	5	4	3	3	4	5	4	2	3
	Item 19	5	5	1	1	4	5	6	1	1	4	5	5	1	1	4	1	1	1	6	3	1	1	1	1	6	5	6	3	1	5	5	6	3	4	5	5	6	3	4	6
	Item 20	1	1	1	1	1	1	1	1	1	1	1	1	1	1	1	1	1	1	1	1	1	1	1	1	1	1	5	1	1	1	1	5	1	1	1	1	5	1	1	1
	Item 21	1	1	1	1	1	1	1	1	1	1	1	1	1	1	1	1	1	1	5	1	1	1	1	1	1	1	2	1	1	1	1	2	1	1	1	1	2	1	1	1
**Editorial independence**	Item 22	4	4	3	4	3	4	4	3	3	3	4	4	3	4	3	4	4	3	1	1	1	2	1	1	1	7	7	7	7	5	7	7	7	7	5	7	7	7	7	5
	Item 23	1	1	1	1	1	1	1	1	1	1	1	1	1	1	1	1	1	1	1	1	1	1	1	1	1	7	7	7	7	5	7	7	7	7	5	7	7	6	7	5

CPG: clinical practice guideline

*Guidelines were divided into conditions: CPG 1: Knee pain; CPG 2: Neck pain; CPG3: Low back pain; CPG4: Hwa-byung(火病)); CPG5: Low fertility; CPG6: Lumbar herninated intervertebral disc; CPG7: Atopic dermatitis; CPG8: Bell's palsy
